# Upper airway obstruction and dyspnea in an infant caused by mediastinal and endotracheal lipoblastoma: a case report

**DOI:** 10.3389/fonc.2025.1535594

**Published:** 2025-03-17

**Authors:** Yulin Chen, Li Qiu, Wei Kou, Zhongqiang Liu

**Affiliations:** ^1^ Department of Pediatrics, West China Second University Hospital, Sichuan University, Chengdu, China; ^2^ Key Laboratory of Birth Defects and Related Diseases of Women and Children of the Ministry of Education, Sichuan University, Chengdu, China; ^3^ Department of Otolaryngology, West China Second University Hospital, Sichuan University, Chengdu, China

**Keywords:** lipoblastoma, mediastinal and endotracheal mass, MRI, airway obstruction, children

## Abstract

**Background:**

Lipoblastoma is a benign tumor of embryonal fat tissue that typically affects children under the age of 3 years. The impact of lipoblastoma on the body largely depends on the tumor’s location and its involvement with adjacent organs. Mediastinal lipoblastoma, a relatively rare form, often presents with dyspnea and difficulty breathing, however, airway involvement has not been previously reported.

**Case presentation:**

We describe a 4-month-old female infant diagnosed with lipoblastoma in the mediastinum and endotracheal region, leading to progressive upper airway obstruction and dyspnea. Diagnosis was confirmed through imaging and histopathological analysis. The patient underwent a series of treatments, including bronchoscopic-guided endotracheal intubation with ventilator-assisted ventilation, ECMO-supported mass resection, and airway reconstruction. The patient ultimately achieved complete recovery, with no tumor recurrence observed at a 2-month follow-up.

**Conclusion:**

Mediastinal lipoblastoma can be a significant cause of upper respiratory obstruction and respiratory distress in children. The potential for tracheal infiltration, as seen in this case, has not been previously reported and can pose a life-threatening risk. Recognizing and understanding this rare presentation is essential for timely diagnosis and effective management of this condition.

## Introduction

Lipoblastomas (LPBs) are rare, benign mesenchymal tumors originating from embryonic fat cells, typically occurring in infancy and early childhood. They are classified into two morphological forms: lipoblastoma and lipoblastomatosis (1). These tumors most frequently occur in the limbs and trunk, with only limited cases reported in the mediastinum (2, 3). To date, no cases of LPB have been reported exclusively within the mediastinum or the tracheal lumen, except for a single case involving both the neck and trachea (4). When located in the mediastinum, LPB can pose a significant risk of airway obstruction, often presenting with symptoms such as dyspnea, cough, and palpitations, which may lead to life-threatening complications. Complete surgical resection is critical to prevent recurrence. Recognizing LPB in these atypical locations is crucial for timely diagnosis and effective management. In this paper, we report a rare pediatric case of upper airway obstruction and dyspnea caused by mediastinal and endotracheal LPB, accompanied by a literature review to improve awareness and management strategies for pediatricians treating LPB.

## Case report

**Figure 1:**
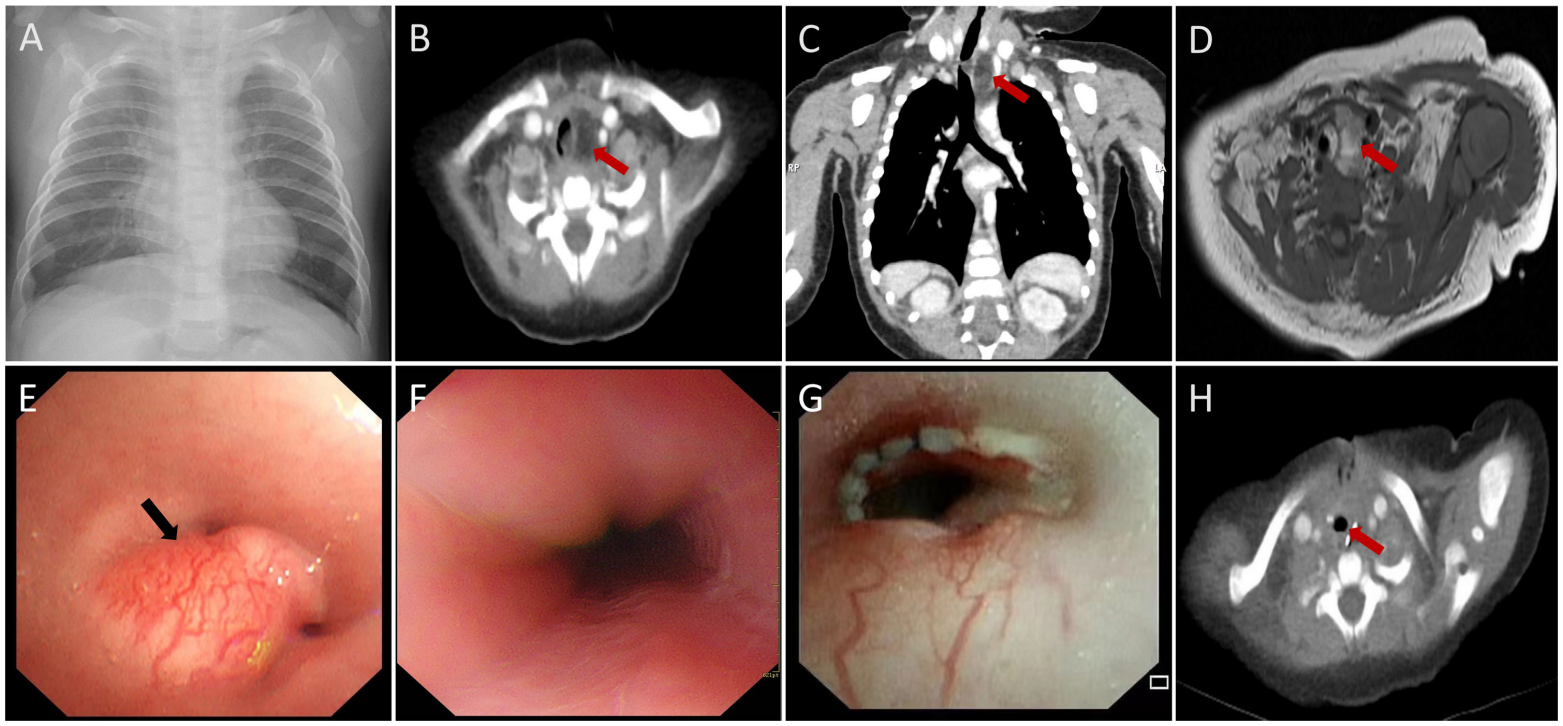
Imaging of mediastinal and endotracheal mass **(A)** Chest radiography demonstrated no obvious mediastinal mass. **(B-D)** CT and MRI revealed a mediastinal and endotracheal fatty mass measuring 1.4×1.2×2.3 cm. The trachea was markedly compressed, and the mass protruded into the tracheal lumen, resulting in significant tracheal stenosis (red arrows). **(E)** Bronchoscopic examination revealed a smooth-surfaced obstructing mass in the upper segments of the tracheal lumen, surrounded by prominent blood vessels (black arrow). **(F)** Esophageal examination showed no abnormalities. **(G, H)** MRI scans taken two months postoperatively and postoperative bronchoscopy revealed no recurrence of the mass (red arrows).

**Figure 2:**
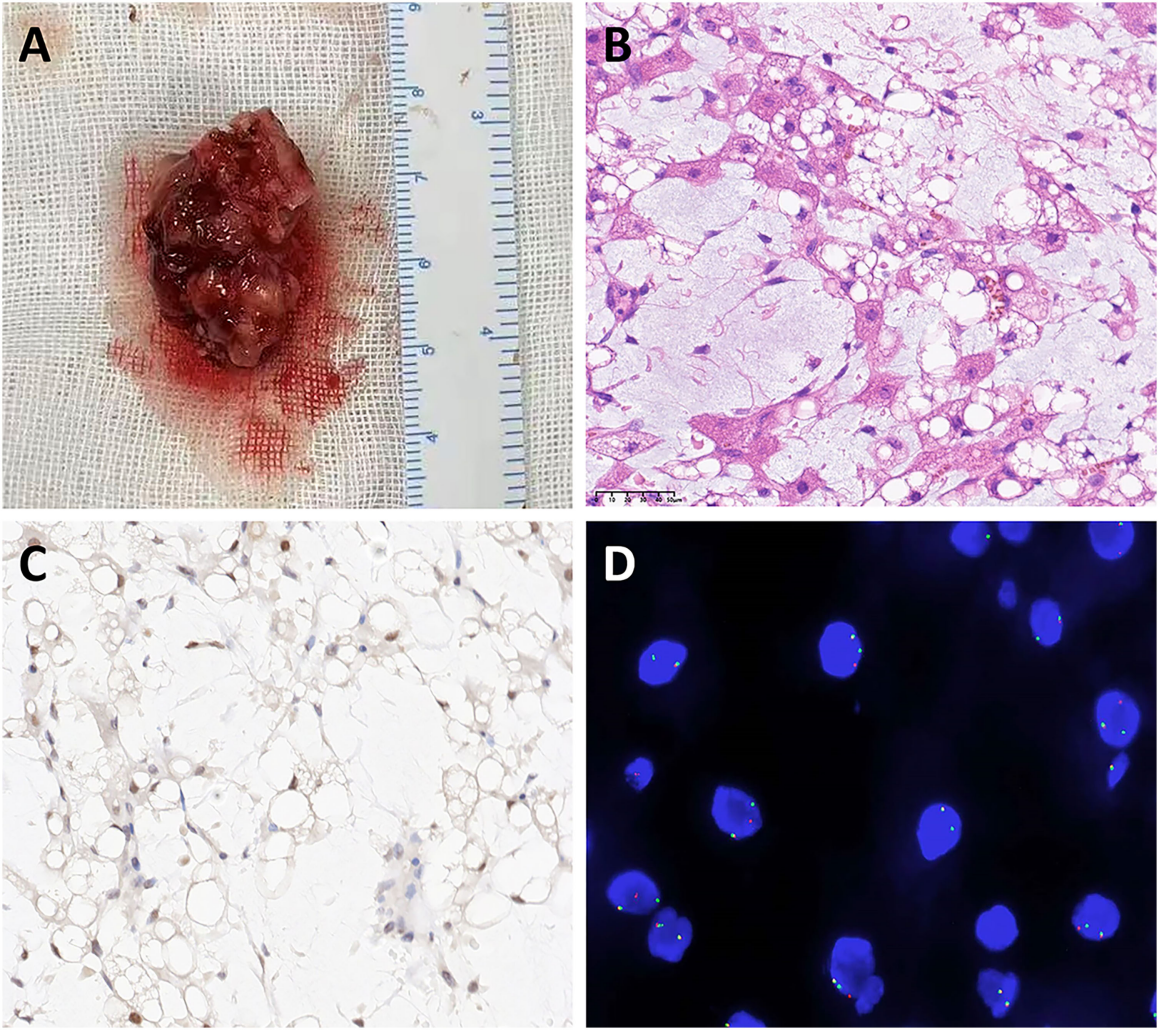
Pathological findings of the mass **(A)** Macroscopic view of a lobular mass with a myxoid appearance measuring 2.5×1.8×0.8 cm. **(B)** Microphotograph (hematoxylin and eosin stain, ×40) demonstrating mature adipose tissue arranged in lobules. **(C)** Immunohistochemical staining showed that the tumor cells were negative for MDM2 (×40). **(D)** FISH analysis detected PLAG1 breakage and rearrangement using a two-color separation probe in lipoblastoma.

A 4-month-old girl was admitted to our hospital with a persistent cough, stridor, nausea, and difficulty breathing persisting for one month. The patient had no significant medical history. A chest X-ray revealed only mild exudative changes (**Figure 1A**). Despite aggressive anti-infective therapy, her respiratory distress worsened. Upon admission to the Pediatric Intensive Care Unit (PICU), her initial vital signs were a heart rate of 165 beats per minute, a temperature of 36.6°C, a respiratory rate of 62 breaths per minute, and an oxygen saturation of 91% while on supplemental oxygen via nasal cannula. Physical examination revealed inspiratory stridor without cyanosis, paraparesis, or hemiparesis. Routine laboratory tests, including a complete blood count, C-reactive protein, and liver function tests, were within normal limits. Chest-enhanced computed tomography (CT) showed a fat-density soft tissue mass in the upper left mediastinum (**Figures 1B, C**) extending into the trachea. Magnetic resonance imaging (MRI) revealed a fat-dominant irregular mass in the same region, with high signal intensity on both T1- and T2-weighted images (**Figure 1D**). Bronchoscopy identified a smooth-surfaced obstructive mass within the upper tracheal lumen (**Figure 1E**). An esophageal examination found no abnormalities (**Figure 1F**). Screening tests for tuberculosis, TORCH infections, neuron-specific enolase, hepatitis viruses, β-human chorionic gonadotropin, alpha-fetoprotein, and bacterial and fungal cultures of the lavage fluid were negative. The patient was initially managed with non-invasive ventilation to correct hypoxia, with the ventilator settings including a positive end-expiratory pressure (PEEP) of 5 mmHg, peak inspiratory pressure (PIP) of 25 mmHg, and a fraction of inspired oxygen (FiO_2_) at 60%. However, despite these interventions, the patient experienced several episodes of cardiopulmonary arrest, which were temporarily resolved with emergency positive-pressure oxygenation to restore the heart rate. Given the recurrent episodes of respiratory distress, bradycardia, and decreased oxygen saturation, the decision was made to proceed with bronchoscopic-guided endotracheal intubation. Anesthesia induction was achieved with propofol at a dose of 2.5 mg/kg. Subsequently, invasive mechanical ventilation was initiated to provide more stable respiratory support. Meanwhile, Extracorporeal Membrane Oxygenation (ECMO) was kept on standby as a resuscitation option in case of further deterioration. After preoperative evaluation and confirmation of the lesion’s location, the patient underwent surgical resection and airway reconstruction with the support of ECMO. During the surgery, a soft tissue mass measuring 2.5×1.8×0.8 cm was identified (**Figure 2A**), which had encased the airway. Due to the infiltration of the tumor into the tracheal tissue, a combined resection of the tracheal segments along with the involved tracheal cartilage was performed, followed by end-to-end anastomosis. The entire surgical procedure lasted for 107 minutes. Postoperatively, the patient was successfully weaned off ECMO and continued on mechanical ventilation. The patient’s heart rate, oxygenation, and blood pressure were all maintained within normal limits. Histopathological analysis of the mass confirmed a diagnosis of lipoblastoma, with no encapsulated borders (**Figures 2A, B**). Immunohistochemistry revealed S-100 positivity in adipocytic cells, while MDM2 and P16 staining were negative (**Figure 2C**). Fluorescence *in situ* hybridization (FISH) analysis showed rearrangement of the pleomorphic adenoma gene 1 (PLAG1) (**Figure 2D**), supporting a final diagnosis of LPB. Given the tumor’s lack of encapsulation and its infiltrative growth pattern, it was more consistent with lipoblastomatosis, a rarer form of lipoblastoma, as described in the literature (1). The postoperative course was uneventful, and the patient did not require further ventilatory support. Bronchoscopy showed no residual foreign body (**Figure 1G**). At a two-month follow-up, there was no evidence of tumor recurrence (**Figure 1H**), and the patient remains under regular surveillance.

## Discussion

LPB predominantly affects children under three years of age (2), with mediastinal LPB cases being rare in pediatric populations (5). There are no documented cases involving both the mediastinum and intraluminal trachea, although one report describes combined endotracheal and neck LPB in a 7-month-old girl (4). To our knowledge, this is the first documented case of LPB concurrently in mediastinum and trachea. It can be life-threatening due to local compression (6), especially when the tumor extends into the trachea. In our patient, the mass extended into the tracheal lumen, resulting in localized stenosis, which caused severe dyspnea and respiratory failure. This case is noteworthy not only due to its rarity but also due to the complexity presented in such a young patient.

Although extremely rare, mediastinal and endotracheal LPB should be considered in patients with recurrent cough or stridor that fails to respond to standard treatments. In this case, the patient’s symptoms of stridor, respiratory failure, and recurrent cardiopulmonary arrest during non-invasive mechanical ventilation raised initial clinical suspicion. Early imaging is essential for diagnosis. Muraoka et al. (7) found that 80% of LPB cases demonstrated abnormal chest radiographic findings, underscoring the value of timely detection through X-rays. However, chest radiography alone cannot accurately locate or measure the mass (8). As observed in our patient, early chest X-rays revealed no masses or space-occupying lesions. While ultrasound can aid in diagnosing LPB, it is less effective in assessing mediastinal or endotracheal involvement. CT and MRI are the preferred imaging modalities, offering comprehensive information on the tumor’s location, size, composition, and relationship to surrounding structures, which is critical for surgical planning.

Due to the high risk associated with tracheal intubation under general anesthesia, and given the likelihood of airway obstruction and the risk of rupture and bleeding caused by the bronchial mass, endotracheal intubation was performed under bronchoscopic guidance. This approach ensured effective respiratory management, emphasizing the importance of bronchoscopy-guided tracheal intubation and ventilation in the prompt and systematic treatment of LPB with such critical symptoms. In our case, fine-needle biopsy was considered inappropriate for diagnosing lipoblastoma due to the significant risk of non-representative sampling and the potential for substantial bleeding. Therefore, this is not recommended. Given the high-risk location of the mass, involving the endotracheal space and extending into the mediastinum, we opted to perform a thoracotomy for definitive management.

The pathogenesis of LPB involves amplification and rearrangement of chromosome 8q11-13, often accompanied by involvement of the PLAG1 gene, which serves as a surrogate marker for PLAG1 fusions commonly seen in LPB (9). MDM2 is typically positive in liposarcomas but negative in LPB (2, 10), aiding in the differential diagnosis. Considering the patient was only 4 months old, the possibility of malignancy in the endotracheal mass could not be excluded due to its invasive growth. For this patient, with elevating levels of human chorionic gonadotropin and α-fetoprotein, and other further screenings were all negative, which helps rule out malignancy, the final definitive diagnosis relied on histopathological analysis, that immunohistochemical staining showed that the tumor cells were negative for MDM2 and FISH analysis detected PLAG1 breakage and rearrangement. Histopathological analysis revealed a non-encapsulated adipose tissue border with an infiltrative growth pattern. Therefore, although imaging and histopathology confirmed LPB, the findings indicate that lipoblastomatosis, a rare variant, may be a more accurate classification for this case. Similar to our case, both Safavi et al. (11) and de Carvalho et al. (12) have reported cases of mediastinal lipoblastoma causing local airway compression. However, neither study described the infiltrative growth of the tumor, especially into the tracheal tissue.

Besides, surgery remains the primary treatment for LPB to preserve vital structures. Complete resection leads to a lower recurrence rate than partial resection (11, 12). Long-term follow-up for 5 to 10 years is recommended due to the risk of late recurrence (13). In our case, no recurrence was observed at the two-month postoperative follow-up, and the patient did not require additional respiratory support.

## Conclusion

Mediastinal and endotracheal LPB should be considered in pediatric patients presenting with upper airway obstruction and dyspnea. Lipoblastomatosis, especially in its infiltrative form, is a rare benign tumor that can be invasive. Due to the atypical clinical presentation and imaging characteristics, lipoblastomatosis is often overlooked or misdiagnosed. An accurate diagnosis requires a combination of MRI, histopathological analysis, and immunohistochemistry. The preferred treatment for LPB is complete surgical excision. Additionally, close postoperative follow-up recommended monitoring for possible recurrence.

## Data Availability

The original contributions presented in the study are included in the article/supplementary material. Further inquiries can be directed to the corresponding author.
